# Quantitative Proteomic Analysis of Human Seminal Plasma from Normozoospermic and Asthenozoospermic Individuals

**DOI:** 10.1155/2019/2735038

**Published:** 2019-03-10

**Authors:** Yibo Wu, Yan Yuan, Ling Chen, Min Wang, Yong Yang, Yingnan Wang, Chao Quan, Daozhen Chen, Ying Chen, Xiaoyan Huang, Tao Zhou

**Affiliations:** ^1^Human Reproductive and Genetic Center, Affiliated Hospital of Jiangnan University, Wuxi 214000, China; ^2^State Key Laboratory of Reproductive Medicine, Department of Histology and Embryology, Nanjing Medical University, Nanjing 211166, China; ^3^Laboratory of Genomic and Precision Medicine, Jiangnan University, Wuxi 214000, China; ^4^Centre for Reproductive Medicine, The Affiliated Wuxi Maternity and Child Health Care Hospital of Nanjing Medical University, Wuxi 214002, China; ^5^Central Laboratory, The Affiliated Wuxi Maternity and Child Health Care Hospital of Nanjing Medical University, Wuxi 214000, China

## Abstract

Seminal plasma is a complex mixture of secretions from various glands in the male genital tract. Compared to sperm cells, it contains important proteins that are both directly and indirectly associated with sperm motility. Here, we constructed quantitative proteomes of human seminal plasma from three normozoospermic and asthenozoospermic individuals. A total of 524 proteins were identified, and 366 of them were found to be quantified in all six samples. We first investigated the absolute expression features of these proteins and found that the variations of protein identification among different samples and other published datasets were mainly due to some lowly expressed proteins. By integration of various proteomic datasets and bioinformatics databases, we comprehensively annotated the biological functions, physiological originations, and disease associations of these proteins. We found that our dataset could benefit the studies of both male infertility and other male diseases. Finally, based on the relative expression values determined by chemical labeling, we identified a total of 29 differentially expressed proteins, which could be used as candidate targets for studying the molecular bases of sperm motility or developing precise diagnostic biomarkers of asthenozoospermia. We further successfully verified the expression trends of four representative proteins by Western blotting. Compared to a previous dataset based on label-free quantification, our results showed that most of the important proteins could be identified in the sample collected only once for each individual, providing the bases for personalized examination of seminal plasma proteins in clinic.

## 1. Introduction

Male factors contribute directly or indirectly to nearly 50% of infertility cases [1]. Based on semen analyses, the most prevalent pathological characteristic of male infertility is asthenozoospermia (low sperm motility), which is responsible for about 81% of cases [2]. There are two main parts of semen: seminal plasma and sperm cells. Mature sperm is a specialized haploid cell with elongated structure and is almost silenced at both transcriptional and translational levels [3]. Sperm functions are tightly regulated by the existing proteins, which have served as a resource pool for screening of key targets involved in regulating sperm motility. In the ejaculated semen, seminal plasma is the fluid surrounding sperm cells. It contains mixed secretions from various glands or tissues in the male genital tract, such as seminal vesicle, prostate, testis, epididymis, and periurethral glands. Thus, seminal plasma is a collection of complex molecules, which plays important roles in sperm maturation, sperm metabolism, sperm motility and sperm capacitation, semen coagulation, semen liquefaction, and fertilization [4]. Compared to sperm cells, seminal plasma could provide more comprehensive resources for discovering key molecules in regulating sperm functions as well as developing biomarkers of male infertility. Since seminal plasma also contains epididymal secretory sperm-located proteins [5], it could provide potential protein targets that are directly or indirectly associated with sperm motility.

Quantitative proteomic approach has been widely used in the field of male reproduction to screen for key proteins for spermatogenesis and male fertility [6]. There are two main proteomic strategies for identifying differentially expressed proteins: label-free (based on spectra counting or peptide intensities) and labeling (based on chemical reagents) methods. Although label-free strategy is more economical and could quantify more proteins, the main drawback is that it is less reliable than labeling methods in determining differential expression [7]. Using Tandem mass tag (TMT) reagents, a recent study has already identified differentially expressed (DE) proteins in sperm samples between asthenozoospermic and normozoospermic groups [8]. There were two previously published proteomic datasets of seminal plasma proteins, which also aimed to identify DE proteins between asthenozoospermic and normozoospermic samples [9, 10]. However, these datasets were all based on label-free methods, which were needed to be verified or improved by a more confident method.

In the present study, we aimed to quantify seminal plasma proteins in individual samples and identify DE proteins between asthenozoospermic and normozoospermic groups using TMT based quantitative proteomic strategy. In addition to relative expression, we also investigated the absolute expression features of all seminal plasma proteins. By integration of various proteomic datasets and bioinformatics databases, we comprehensively annotated the biological functions, physiological originations, and disease associations of these proteins, which could benefit the studies of both male infertility and other male diseases.

## 2. Materials and Methods

### 2.1. Ethics Statement and Sample Collection

The project was approved by the Ethics Committee of the Affiliated Hospital of Jiangnan University. All methods were performed in accordance with the relevant guidelines and regulations. Written informed consents were obtained from all enrolled volunteers (recruited from local residents in Wuxi) prior to sample collection. All clinical information (including basic information and diagnostic results) from volunteers is anonymized. As a basic research, the current results do not provide direct guidance for the diagnosis and treatment of male infertility.

Semen samples were obtained by masturbation into sterile containers after about 5 days of abstinence. Normozoospermia (progressively motile sperm >40%) and asthenozoospermia (progressively motile sperm <32%) were mainly diagnosed and distinguished based on sperm motility, according to World Health Organization (WHO) 2010 criteria. To avoid ambiguous factors, severe teratozoospermic and necrospermic samples were excluded. The age of donors and the total volume of semen were also required to be similar between normozoospermic and asthenozoospermic groups. For proteomic analysis, semen samples from three donors were selected for each group. The samples were further divided into two parts for technical repetitions. Six other samples for each group were newly collected for two rounds of Western blotting analysis. The detailed semen parameters for each sample, including volume, cell number, percentage of motile sperm, and seminal specific indicators (such as liquefaction time, pH, alpha-glucosidase, zinc, and fructose concentrations), are listed in Supplementary Data 1.

### 2.2. Protein Extraction, Digestion, and TMT Labeling

To avoid nonspecific proteolysis during liquefaction and protein extraction, a cocktail of proteases inhibitors (Pierce, Rockford, USA) was added to seminal plasma within few minutes after ejaculation. Each semen sample was liquefied for 30 minutes at 37°C and then was centrifuged at 40,000g for 30 minutes at 4°C to remove cell debris and other impurities. Protein concentration was assessed using the Bradford method. Proportionally, the liquid with 2mg proteins was reduced in 20*μ*l 1M dithiothreitol (DTT) at 56°C for one hour and then treated with 100*μ*l 1M iodoacetamide (IAA) in the dark for 45 minutes. After that, 7 times of the total liquid volume of acetone solution buffer were added overnight at -20°C to precipitate proteins. After centrifugation at 8000g for 10 minutes at 4°C, the precipitates were dissolved with urea (8M urea, 75mM NaCl, 50mM Tris, pH 8.2).

For protein digestion, the sample was treated with DTT to 5mM for 25 minutes at 56°C and then with iodoacetamide to 14mM for 30 minutes at room temperature in the dark, and DTT was added to 5mM for 15 minutes at room temperature in the dark. The protein mixture was diluted 1:6 in 25mM Tris-HCl, pH 8.2. Subsequently, 10*μ*g trypsin (Promega, WI, USA) and 1mM CaCl_2_ were added overnight at 37°C in a shaker. The digestion was stopped by acidification with FA (formic acid) to 1% (vol/vol).

Tandem mass tag (TMT) 6-plex isotopic label reagent (Thermo Scientific, Rockford, USA) was used for peptide labeling, according to the manufacturer's instructions. In brief, the reagents were first equilibrated to room temperature. Each aliquot was resuspended in 41*μ*l of anhydrous acetonitrile and then added to the corresponding peptide sample (equal to 100 ug proteins) dissolved in 200mM triethylammonium bicarbonate (TEAB). After 60 min of reaction at room temperature, 8 *μ*l hydroxylamine 5% (w/v) was added and incubated for 15 min. Three asthenozoospermic samples were labeled with TMT-126, TMT-127, and TMT-128, while three normozoospermic samples were labeled with TMT-129, TMT-130, and TMT-131, respectively. Finally, all six aliquots were combined for the following mass spectrometry analysis.

### 2.3. LC-MS/MS Analysis

The peptide mixture was analyzed using a LTQ Orbitrap Velos mass spectrometer (Thermo Fisher Scientific, San Jose, CA) combined with an LC system. In brief, samples were first loaded onto a *μ*-precolumn cartridge (0.3×5mm, 5*μ*m, 100 Å; DIONEX, Sunnyvale, CA) at a flow rate of 20 *μ*l/min. The following experimental procedure and detailed parameters for LC transfer and separation followed our previous publication [11].

MS analysis was performed in data-dependent acquisition mode. An MS survey scan was obtained for the m/z range of 400-1800 at a resolution of 60,000. A low-energy MS/MS scan of every precursor in the linear ion trap (collision induced dissociation, CID) followed by a higher energy MS/MS scan in the octopole collision cell (higher energy collision dissociation, HCD) was acquired from the survey scan for the eight most intense ions (as determined by Xcalibur mass spectrometer software in real time). Dynamic mass exclusion windows and lock mass used were also the same as described previously [11].

### 2.4. Protein Identification and Quantification

The raw files were first converted into mzXML files, and then the paired CID/HCD spectra were merged using in-house developed scripts. The reconstructed mzXML files were searched against the UniProt reference proteome of human (release: 2018_02) [12] using MaxQuant software (version: 1.5.2.8) [13]. Enzyme specificity was set to be fully cleaved by trypsin, and the maximum number of missed cleavage sites permitted was two. The mass tolerance for precursor ions was set to 20 ppm at the first search as applied in MaxQuant for initial mass recalibration. For the main search, the mass tolerance for precursor ions was set to 6 ppm. The mass tolerance for fragment ions was set to 0.5 Da. In addition to TMT reagent adducts (+229.162932 Da) on lysine and the N-terminus of peptides, Carbamidomethyl (+57.02146 Da) on cysteine was set as a fixed modification, while oxidation (+15.99492 Da) on methionine and acetylation (+42.01056 Da) on the N-terminus of proteins were set as variable modifications. The minimum peptide length required was six amino acids, and at least one unique peptide was required for each protein group. The false discovery rate (FDR) of identification was estimated by searching a reversed sequence database. The FDRs for peptide and protein were all set to 0.01.

The absolute expression level of different proteins was estimated using the iBAQ (Intensity Based Absolute Quantification) algorithm embedded in MaxQuant. The identification results by MaxQuant were further optimized using the automatic reporting tool, MaxReport (version: 2.2) [14]. The relative expression values for each protein among different samples were calculated by a modified Libra algorithm embedded in MaxReport. For the identification of differentially expressed (DE) proteins between normozoospermic and asthenozoospermic groups, expression values of all unique peptides for each protein were compared based on the intensities of the labeled reporter ions correspondingly. The criteria for statistical significance were based on a combination of P value (less than 0.05, unpaired Student's* t*-test) and fold change (larger than 1.5) strategy as described previously [15].

### 2.5. Bioinformatics Analysis

Protein IDs were converted to Ensembl gene IDs to make it more convenient to compare the results of different datasets. The mapped Ensembl gene IDs were also used to perform functional annotation. The ToppGene suite [16], which integrated various bioinformatics databases including Gene Ontology, human disease, and mouse phenotype, was applied to investigate the biological and disease associations of seminal plasma proteins. For functional enrichment analysis, the human genome was set as the background and an adjusted P value by the Benjamini & Hochberg (BH) method less than 0.05 was considered as statistically significant. The Cytoscape software (version: 3.2) was applied to generate visualized networks of relations between gene and functional terms [17].

### 2.6. Western Blot Analysis

Seminal plasma proteins were extracted from three different asthenozoospermic and three normozoospermic specimens, as described above (using the same cocktail of proteases inhibitors). The basic procedure of Western blotting used was described previously [18]. Membranes were incubated with the following primary antibodies: 1:1000 anti-KLK2 (rabbit polyclonal, SAB2104725, Sigma Aldrich, USA), 1:5000 anti-HSPA2 (rabbit polyclonal, ab154374, Abcam, Hong Kong, China), 1:2000 anti-SORD (rabbit polyclonal, ab185705, Abcam, Hong Kong, China), 1:2000 anti-ANXA2 (rabbit polyclonal, ab41803, Abcam, Hong Kong, China), and 1:1000 anti-beta Tubulin (rabbit polyclonal, ab6046, Abcam, Hong Kong, China). The whole experiment was repeated using another group of samples obtained from six different individuals.

## 3. Results and Discussion

### 3.1. Individualized Proteomic Profiling of Human Seminal Plasma

**Figure 1:**
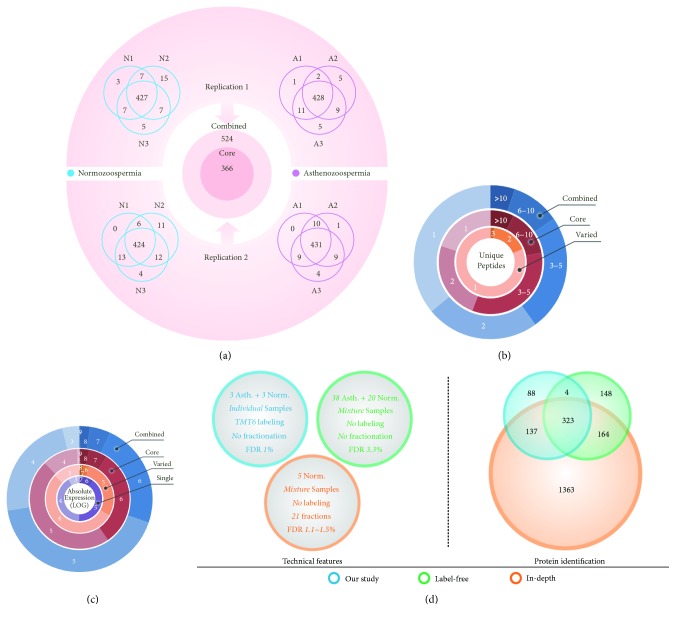
*Summary of the individualized proteomes of human seminal plasma*. (a) Comparison of protein identification among six individuals. (b) Distribution of unique peptides. (c) Distribution of absolute expression levels. (d) Comparison of the technical features and protein number among different datasets.

Using TMT 6-plex reagent, we were able to identify protein compositions from six individual samples in a single MS experiment. Three asthenozoospermic (A1, A2, and A3) and three normozoospermic (N1, N2, and N3) subjects were enrolled. Seminal plasma sample was only collected once for each individual. Two experimental repetitions were performed to evaluate technical variation. On average, more than 440 proteins were identified in each individual sample (Figure 1(a); Supplementary Data 2). Combining the results of two technical repetitions, a total of 524 proteins (the combined list) were identified, of which 366 (the core list) proteins were ubiquitously quantified in all samples and repetitions, according to the intensities of reporter ions. However, among each group, more than 90% of proteins could be quantified in all three samples. Only 21 proteins (experimental variation) were specific to normozoospermic or asthenozoospermic group, while 116 proteins were only quantified in one repetition for each group (technical variation). The above-mentioned two classes of varied proteins were also combined and termed as the varied list.

We then compared the distributions of unique peptide number among different protein lists. About 36% and 20% of proteins were identified with only one unique peptide for the combined and core list, respectively, while about 81% of proteins in varied list were identified with one unique peptide (Figure 1(b)). The intensities of reporter ions were mainly used for relative expression comparison (expression levels between different groups). We further applied the iBAQ algorithm to calculate the average absolute expression values for every gene in each repetition. According to iBAQ intensities, these proteins cover nearly seven orders of magnitude (from 10^3^ to 10^9^). The proportions of lowly expressed proteins (10^3^ and 10^4^) in the combined and core lists were obviously smaller than those in the varied and single peptide lists (Supplementary Figure 1). As shown in Figure 1(c), only 13% of proteins were lowly expressed in the core list, while 67% of proteins were lowly expressed in the varied list. Thus, the majority of identification or quantification variation could be explained by the existence of lowly expressed proteins or proteins with low coverage (with one unique peptide) in human seminal plasma. Nevertheless, we found that the average iBAQ intensities were highly correlated for those proteins identified in both repetitions (r = 0.97 and* p* < 0.001; Supplementary Figure 2), which indicates that the technical repetitions are highly reproducible.

Several datasets about human seminal plasma proteome have already been published. We chose two representative datasets: the “label-free” [9] and the “in-depth” [19] datasets. We compared the differences of technical features, protein number, and potential biomarker coverage among these datasets (Figure 1(d)). In our study, although all samples were combined in the LC-MS/MS analysis, individual samples can be distinguished from each other according to the corresponding labeling reagents. However, protein compositions in the label-free and in-depth datasets cannot be assigned to each individual due to simple mixing procedure. The in-depth dataset performed peptide prefractionation before MS analysis, while our study and the label-free dataset only applied general LC-MS/MS strategy. Finally, the statistical cut-off for protein identification is also different. Our study applied the most stringent criterion (FDR < 0.01). For protein number, the in-depth dataset identified the largest number of proteins, about 3.5 folds of our study. Although prefractionation strategy could greatly improve the total number of protein identifications [20], recent studies showed that proteomics approach without prefractionation provides a low-cost and high-efficiency strategy for initial screening of biomarkers in plasma [21, 22]. About 83% of proteins in our study were also found in the in-depth dataset, and the corresponding ratio for the label-free dataset was 76%, indicating that the in-depth dataset covers most of the proteins of other datasets. However, only 59% of the proteins in our study were overlapped with the label-free dataset, mainly due to technical and biological variations. The present and the label-free studies did not apply peptide prefractionation procedure to reduce sample complexity and improve coverage, which may result in high variation for different experiments.

### 3.2. Comprehensive Functional Annotation of Seminal Plasma Proteins

**Figure 2:**
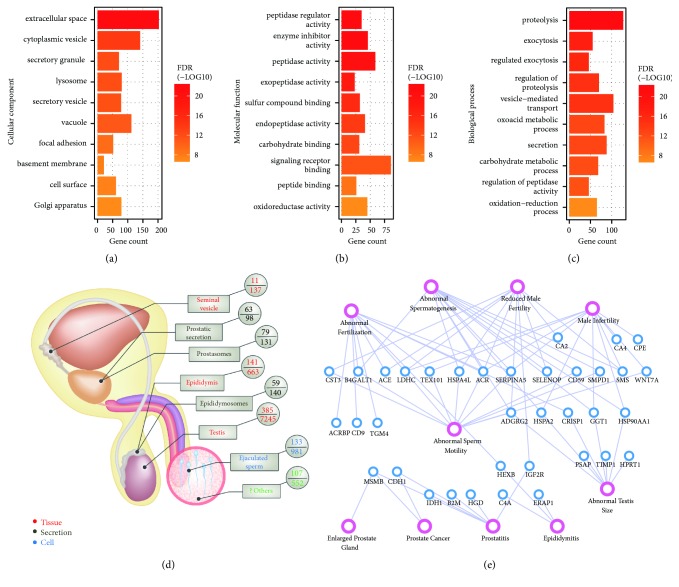
*Bioinformatics annotation of seminal plasma proteins*. The top ten representative functional enriched terms for cellular component (a), molecular function (b), and biological process (c). (d) Mapping of seminal plasma proteins to the tissues (red text), fluids (black text), or cell type (blue text) in the male genital tract. (e) Relation network of proteins (blue circles) and disease associations (pink circles).

Gene Ontology defines detailed functional terms in three main categories: cellular component, molecular function, and biological process. In each GO term, a group of genes with the same function are listed together [6]. We then performed GO enrichment analysis to obtain an overview of the biological associations of the seminal plasma proteins identified in our study. In summary, 411, 419, and 382 proteins were assigned to functional terms in the categories of cellular component, molecular function, and biological process, respectively (Supplementary Data 2). As expected, most of the enriched terms of subcellular localization were directly or indirectly associated with secreted proteins, such as extracellular space and secretory vesicle (Figure 2(a); Supplementary Data 3). About 46% of proteins were annotated to be localized in extracellular space. GO annotations may contain evidences derived from bioinformatics prediction. We further searched the manually checked “Subcellular location” evidences at protein level obtained from UniProt. Among the 301 annotated proteins, 141 proteins were found to be secreted (Supplementary Data 2). The proportion of secreted proteins (47%) is highly consistent with GO annotation. In a previous proteomic study of human seminal plasma [23], only 52% of proteins were mapped to the GO database due to poor annotation in 2006. Only about 25% of these proteins were annotated as extracellular or secreted proteins. Thus, the present study provides an improved and high-confidence annotation of subcellular localization compared to previous study. Seminal plasma may contain membrane-enveloped secretory vesicles such as prostasomes and epididymosomes [24]. Thus, it is also reasonable to find terms related to vesicle or Golgi apparatus, which are involved in generating exosomes. Interestingly, as exposed to extracellular fluid, some proteins were prone to be located in cell surface, adhesion, or basement membrane. In addition to these enriched localizations, a small part of proteins (about 4%) were located in cytoplasmic region. These proteins may be originated from epithelial shredding. However, these proteins are an inseparable part of seminal plasma and could also serve as a source for biomarker discovery. Thus, we cannot treat them as contaminants and simply rule out these proteins.

For molecular functions, the most significant enriched terms were enzyme related functions, such as peptidase regulator activity and peptidase activity (Figure 2(b)). A total of 146 enzymes were identified in our study (Supplementary Data 2). Various enzymes may play important roles in maintaining both semen characteristics and sperm functions. For example, the semen coagulum is needed to be liquefied by the proteolytic enzyme KLK3, which is also known as prostate-specific antigen (PSA), after ejaculation [25]. Several enzymes were already reported to be associated with semen quality, such as MMP2 (correlates to the sperm count) [26] and ALAD (protects sperm from oxidative damage) [27]. On the other hand, some enzymes are needed to be tightly regulated under specific conditions. Thus, many inhibitors were also found to be overrepresented in seminal plasma proteins. For example, a total of 30 protease inhibitors were identified in our study, including A2M, the major inhibitor of PSA [28]. Other enriched terms may represent the basic molecular activities of these proteins, such as carbohydrate binding and peptide binding. Correlated with the prominent terms of cellular component and molecular functions, seminal plasma proteins were highly involved in biological processes such as proteolysis, secretion, and oxidation-reduction (Figure 2(c)).

Seminal plasma is a complex mixture of secretions from various glands in the male genital tract. To further investigate the potential originations of seminal plasma proteins, we mapped these proteins to previously published proteomes of three tissues (seminal vesicle [29], epididymis [5], and testis [30]), three types of fluids (prostatic secretion [31], prostasomes [32], and epididymosomes [33]), and one cell type (ejaculated sperm [34]) separately (Supplementary Data 2). In summary, 385, 141, 59, 11, 63, 79, and 133 gene products (based on Ensembl gene IDs) were assigned to testis, epididymis, epididymosomes, seminal vesicle, prostatic secretion, prostasomes, and mature sperm, respectively (Figure 2(d)). Combining all overlapped genes, there were still 107 genes that were not mapped to any dataset, partially due to limited technical coverage of some proteomes. It also should be noted that there is currently no data available for some apparatuses (such as periurethral glands) with minor contributions to seminal plasma. It is well known that both testicular milieu and posttesticular secretion play important roles in sperm maturation [35, 36]. Thus, it is not surprising that many proteins were also found in testis, epididymis, or prostatic fluid. However, it is interesting that we found that 133 (24%) sperm genes were overlapped with seminal plasma. We further checked the label-free dataset of seminal plasma as described above [9] and also found 156 (24%) sperm genes (Supplementary Figure 3A). The sperm proteome used for comparison was an old dataset with medium coverage [34]. We further compared the gene products between the integrated datasets of seminal plasma (with 2626 genes) [37] and human sperm proteome (with 6751 genes) [38], both with the largest coverage at present. Surprisingly, 1927 (73%) gene products of seminal plasma were overlapped with sperm (Supplementary Figure 3B). There were three possible reasons for this phenomenon. First, sperm surface contains many proteins derived from epididymal secretion (known as epididymal secretory sperm-located proteins) [5]. Second, seminal plasma may also contain some detached proteins from sperm surface. At last, the presence of many sperm proteins may be coexpressed with gene products between different tissue and cell types. For example, a group of genes are ubiquitously expressed in various tissue and cell types, which are known as housekeeping genes [39]. Actually many proteins were known to be found in body fluids, which result from epithelial shredding [40]. This is known as “protein leakage” phenomenon. We thus searched for housekeeping genes [39] and epididymal secretory sperm-located proteins [5] in our study and found that the number of overlapped genes that are specific to sperm proteome (the medium coverage dataset) was reduced to 68 (Supplementary Figure 3C). Combining the above results, we found that human seminal plasma and sperm share a large number of proteins due to the complex origination of seminal plasma rather than random contaminations.

To further evaluate the potential clinical application of seminal plasma proteins, we searched for known disease associated genes combining the evidences of human and mouse phenotypes [41]. As shown in Figure 2(e), a total of 26 genes were annotated to be associated with phenotypes of male fertility, including abnormal spermatogenesis, abnormal sperm motility, abnormal fertilization, male infertility, and reduced male fertility. In addition, we also found that 17 genes (note that 7 genes are overlapping with male fertility) were associated with various tissue phenotypes of male genital tract, such as abnormal testis, epididymitis, prostatitis, enlarged prostate gland, and prostate cancer. Thus, the present proteomic profiling of human seminal plasma, even with a medium coverage, could provide potential biomarkers for both male fertility and diseases related to male genital tract.

### 3.3. Identification of Differentially Expressed Proteins between Normozoospermia and Asthenozoospermia

Besides the absolute expression information for each protein, the present dataset mainly provided relative expression values between normozoospermic and asthenozoospermic groups. A total of 472 proteins were quantified with TMT labeling in repetitions R1 or R2, and 420 proteins were quantified in both repetitions. The overall expression clustering result showed that there exist sample variations within the same group to some extent, mainly due to the heterogeneous features of seminal plasma between different individuals (Supplementary Figure 4). Using the combined statistical criteria of P value (smaller than 0.05) and fold change (larger than 1.5), a total of 111 and 97 differentially expressed proteins were identified in R1 and R2, respectively. We further required that DE proteins must be consistent in both repetitions and obtained a stringent list of 29 proteins (Supplementary Data 2). Compared to asthenozoospermic group, 22 proteins were upregulated and 7 proteins were downregulated in normozoospermic group. The maximum average fold change was -4.3 for LDHC. However, the overall expression ratios were known to be underestimated due to the intrinsic feature of TMT labeling at MS2 level [42]. Thus, the cutoff value for fold change was set to 1.5 in our study.

**Figure 3:**
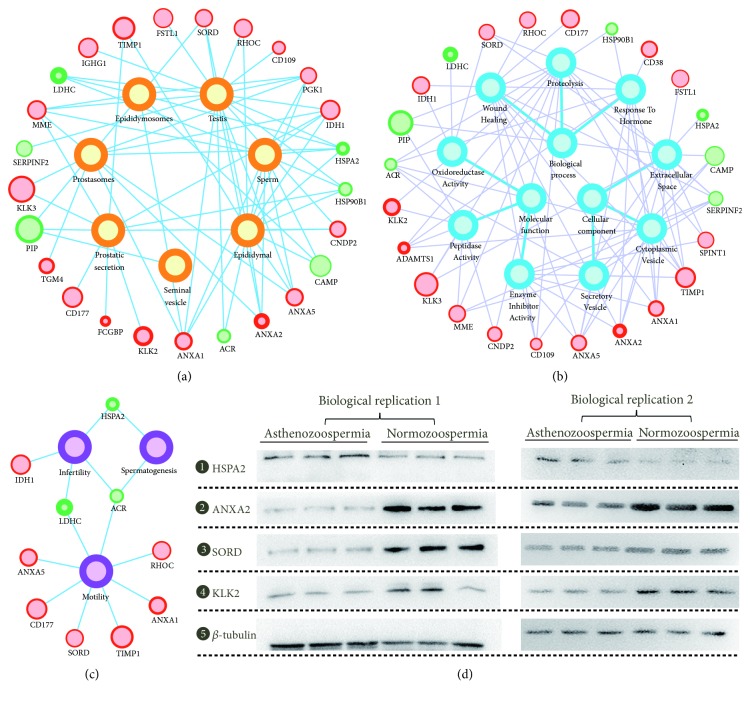
*Annotation and verification of the differentially expressed proteins*. Relation networks for potential originations (a), GO annotations (b), and phenotype associations (c). The inner circle size and the border width represent the absolute and relative expression level, respectively. (d) Western blotting verification of four representative proteins.

Using the above annotated information, we first mapped these DE proteins to the proteomes of male genital tract. As shown in Figure 3(a), a total of 25 proteins were successfully assigned, including 17 testis proteins, 12 epididymal proteins, 11 sperm proteins, 11 prostasome proteins, 10 prostatic secretory proteins, 6 epididymosome proteins, and one seminal vesicle protein. Furthermore, 24 proteins were annotated with GO terms. Similar to the functional enrichment results of all seminal plasma proteins, most DE proteins were located in extracellular space and involved in proteolysis with peptidase or inhibitor activities (Figure 3(b)). As shown in Figure 3(c), 10 proteins were known to be associated with sperm functions or male fertility, including motility (8 proteins), infertility (3 proteins), and spermatogenesis (2 proteins). For example, LDHC (lactate dehydrogenase C) is a canonical testis-specific enzyme and is known to be required for normal male fertility [43]. The expression of LDHC was verified to be greatly decreased in nonobstructive azoospermia by multiplex selected reaction monitoring assay [44]. In our study, we found that LDHC was decreased in asthenozoospermic group with the largest fold change, suggesting that it could also be used as a candidate biomarker for asthenozoospermia. Another example is SORD (sorbitol dehydrogenase), which is involved in energy production via converting sorbitol to fructose. Using mouse model, SORD was found to play a role in driving sperm motility and protein tyrosine phosphorylation [45]. In our study, SORD was elevated in normozoospermia, suggesting that it could be used as an indicator for good sperm motility.

Besides these 10 well-studied proteins, we believe that the other DE proteins could also serve as novel targets for regulating sperm motility or candidate biomarkers for male infertility. However, further experiments are needed to verify their functions. And it also should be noted that we only used six individual samples for proteomic analysis due to the limitation of TMT labeling. The development of clinic biomarkers based on proteomic strategies usually undergoes three steps: initial discovery with small samples, verification with medium samples, and final validation with large samples [46]. Thus, our study was only at the outset stage for developing asthenozoospermic biomarkers. In the future, we will expand the sample number and verify these markers using targeted proteomic method based on selected reaction monitoring.

Some clinicians argued that it is meaningless to find protein biomarkers for semen, since physicochemical parameters derived from semen analysis seem to be enough for diagnosis. However, those parameters, such as semen volume, sperm motility, and sperm morphology, are actually physiological phenotypes. It is of vital importance to identify differentially expressed proteins between normozoospermia and asthenozoospermia, which will help us understand the molecular bases for the occurrence of poor sperm motility. The seminal parameters (including liquefaction time, pH, alpha-glucosidase, zinc, and fructose concentrations) of the samples enrolled in our study were not statistically different between normozoospermic and asthenozoospermic group. Thus, these proteins could also be used as potential targets for developing precise diagnostic biomarkers, as a complementary approach to traditional semen analysis. The cause and consequence of asthenozoospermia may be complex in clinic. Protein biomarkers could also be used for precise diagnosis and treatment of asthenozoospermia in the future. One good example is the development of a clinical assay (combines two proteomic biomarkers: ECM1 and TEX101 in seminal plasma) for precise and noninvasive diagnosis of azoospermia [47]. Thus, these DE proteins could serve as candidate targets for studying the molecular bases of sperm motility as well as developing precise biomarkers for the diagnosis and treatment of asthenozoospermia.

Using label-free strategy based on spectral counting or peptide intensities, two previous proteomic studies have already identified candidate DE proteins between asthenozoospermic and normozoospermic groups [9, 10]. Although quantitative proteomic approach based on label-free strategy could quantify more proteins, the accuracy is less reliable than labeling methods [7]. We compared the DE proteins identified in our study to the latest label-free dataset of asthenozoospermia [10]. Most of the DE proteins in our study were quantified in the label-free dataset. However, due to different sample sources, processing procedures, and proteomic methods, the composition and change trend of the DE proteins were largely different. Only 9 proteins were found to have the same change trend with the label-free dataset, and the remaining 13 proteins were found to be opposite (Supplementary Figure 5). We then chose four representative proteins, one protein (KLK2) with the same trend and three proteins (HSPA2, ANXA2, and SORD) with the opposite trend, to verify their expression. Six newly collected asthenozoospermic and normozoospermic samples (obtained from 12 other individuals) were used for two separate Western blotting experiments. The change trends of all four proteins were consistent with the results of TMT labeling (Figure 3(d)). Thus, the DE proteins identified in our study based on TMT labeling method are of high quality.

## 4. Conclusions

In summary, we constructed quantitative proteomes of human seminal plasma from normozoospermic and asthenozoospermic individuals based on TMT labeling method. A total of 524 proteins were identified, 366 of which were ubiquitously quantified in all samples. The variations of protein identification among different samples or datasets were mainly due to some lowly expressed proteins. We then systematically annotated these proteins to provide a comprehensive understanding of their biological functions, potential originations, and disease associations. Functional enrichment results of GO showed that seminal plasma proteins were mostly secreted proteins and mainly involved in enzyme or inhibitor activities. Using previously published proteomes, we assigned these proteins to the tissues, secretions, or cell type in the male genital tract, providing information of coexpression or potential originations for seminal plasma proteins. The results of disease associations indicated that the present dataset could serve as a resource for discovering key genes for both male fertility and other diseases related to male genital tract.

Finally, based on the relative expression values determined by TMT labeling, a total of 29 DE proteins were identified. We further successfully verified the expression trends of four representative DE proteins by Western blotting. Compared to a previous dataset based on label-free quantification, our results showed that most of the important proteins could be identified in a single sample collected only once for each individual, providing the bases for personalized examination of seminal plasma proteins in clinic. Bioinformatics analysis revealed that several DE proteins were known to be highly associated with sperm motility and male infertility, suggesting that these DE proteins could serve as candidate targets for studying the molecular basis of sperm motility or developing precise diagnostic biomarkers of asthenozoospermia. However, as a preliminary study, further verification and validation experiments were needed to screen for confident targets for sperm motility and asthenozoospermia.

## Data Availability

Proteomic quantification results and bioinformatics results are provided as supplementary data. Other datasets generated or analyzed during the current study are available from the corresponding author upon reasonable request.

## Supplementary Materials

Supplementary 1 Supplementary Figure 1. Comparison of the iBAQ values (log-transformed) among different datasets.

Supplementary 2 Supplementary Figure 2. Correlation of the iBAQ values between two technical repetitions.

Supplementary 3 Supplementary Figure 3. Comparison of protein identification between and among different datasets.

Supplementary 4 Supplementary Figure 4. Clustering analysis of all seminal plasma proteins based on labeling intensities.

Supplementary 5 Supplementary Figure 5. Comparison of the change trends between our study and the label-free dataset.

Supplementary 6 Supplementary Data 1. Sample information for proteomic and Western blotting analyses.

Supplementary 7 Supplementary Data 2. Detailed information for all identified seminal plasma proteins (including basic proteomic identification results, quantification results, functional annotation, and dataset comparison for each protein).

Supplementary 8 Supplementary Data 3. Detailed information for functional annotation.
