# Supplementary figures and images for: Quantitative Proteomic Analysis of Human Seminal Plasma from Normozoospermic and Asthenozoospermic Individuals
Source: Biomed Res Int. 2019 Mar 10;2019:2735038. doi: 10.1155/2019/2735038 (PMC6431472; doi:10.1155/2019/2735038)

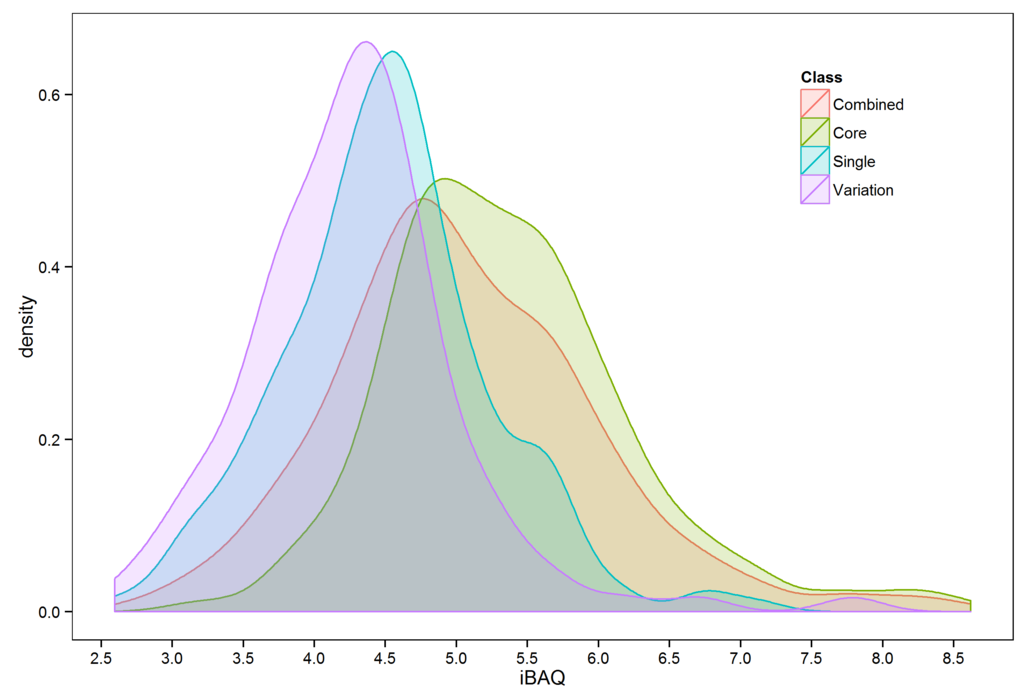

Supplement: Supplementary 1 — Supplementary Figure 1. Comparison of the iBAQ values (log-transformed) among different datasets. [file 2735038.f1.tif]

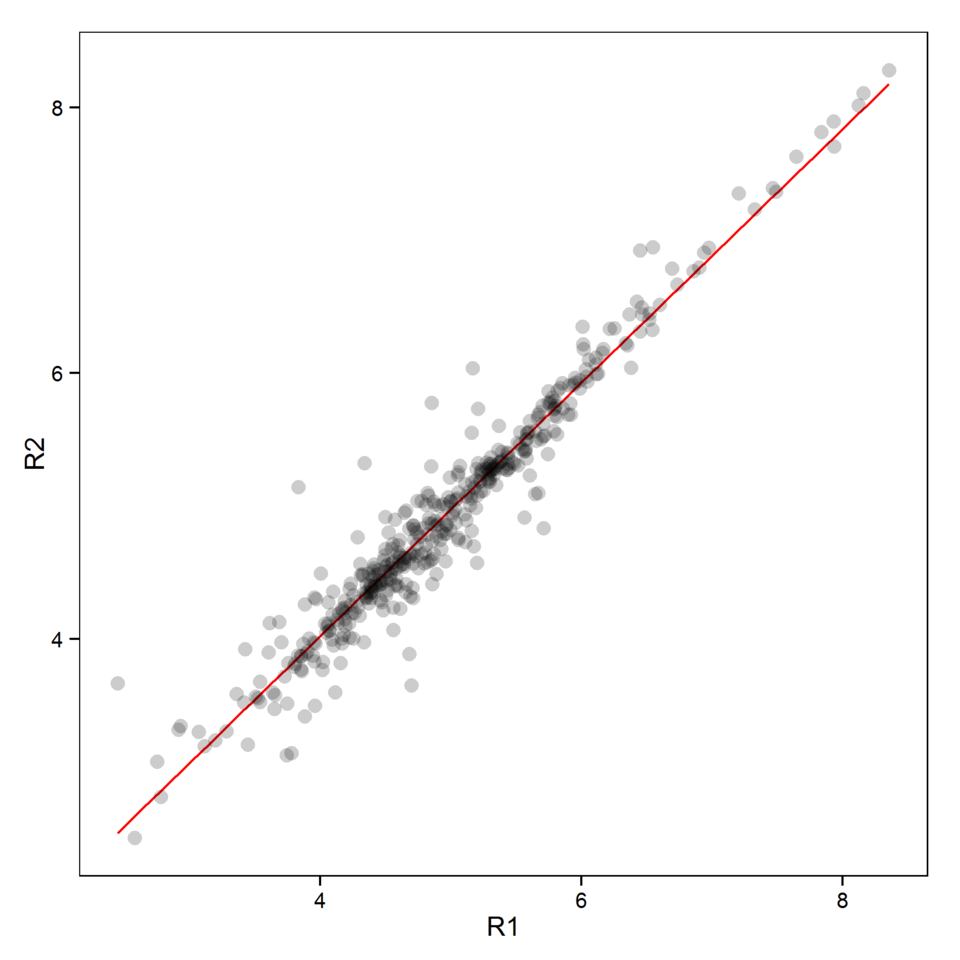

Supplement: Supplementary 2 — Supplementary Figure 2. Correlation of the iBAQ values between two technical repetitions. [file 2735038.f2.tif]

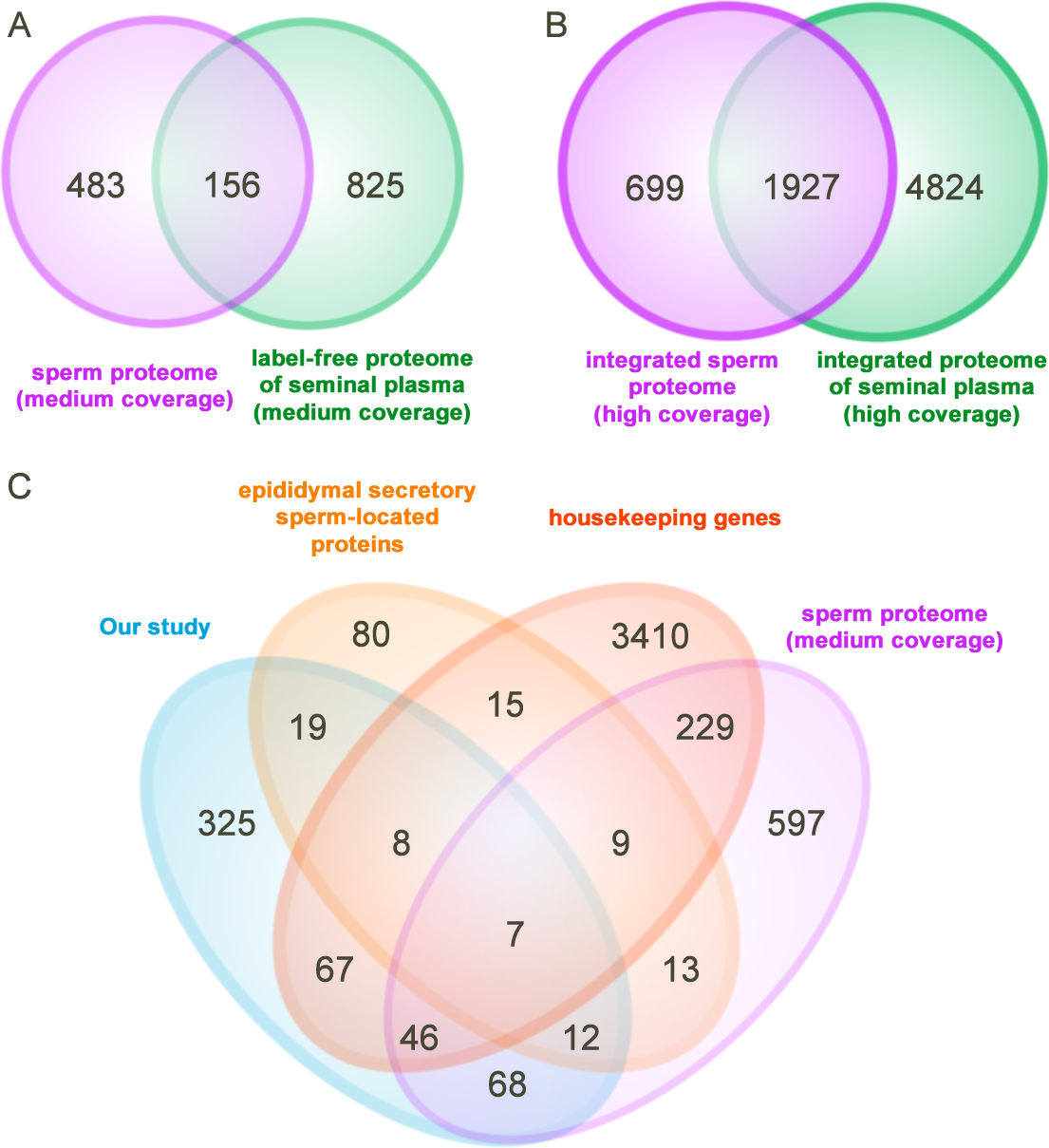

Supplement: Supplementary 3 — Supplementary Figure 3. Comparison of protein identification between and among different datasets. [file 2735038.f3.tif]

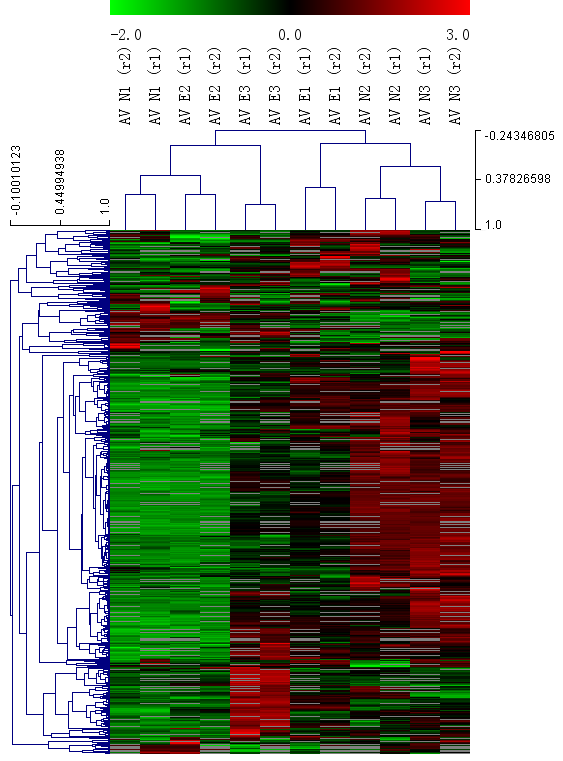

Supplement: Supplementary 4 — Supplementary Figure 4. Clustering analysis of all seminal plasma proteins based on labeling intensities. [file 2735038.f4.tif]

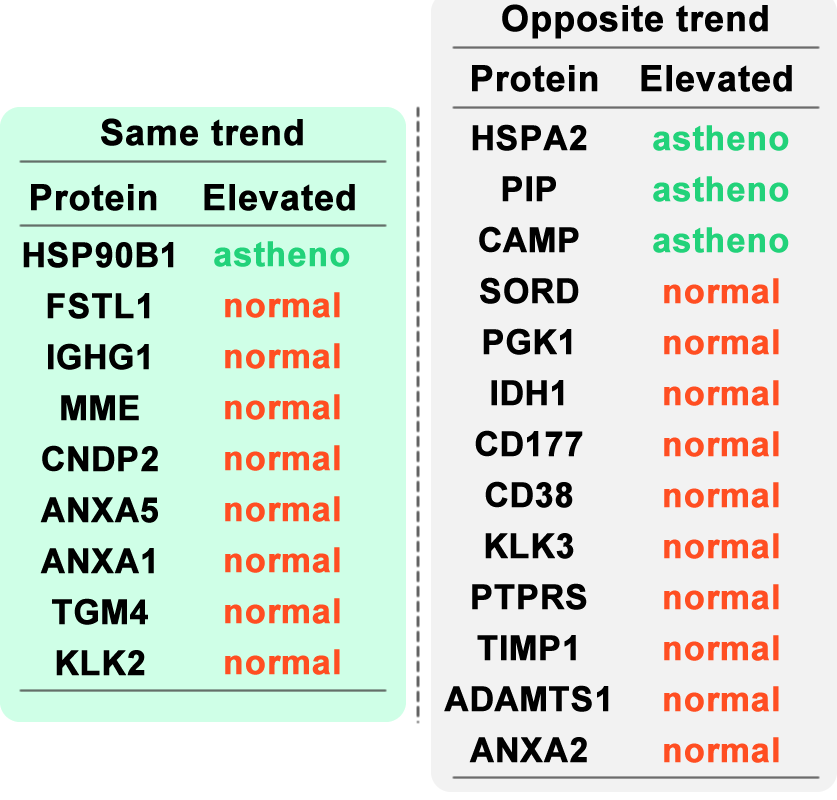

Supplement: Supplementary 5 — Supplementary Figure 5. Comparison of the change trends between our study and the label-free dataset. [file 2735038.f5.tif]
